# Adaptation of an Evidence-Based Arthritis Program for Breast Cancer Survivors on Aromatase Inhibitor Therapy Who Experience Joint Pain

**DOI:** 10.5888/pcd12.140535

**Published:** 2015-06-11

**Authors:** Kirsten A. Nyrop, Leigh F. Callahan, Christine Rini, Mary Altpeter, Betsy Hackney, Arielle Schecher, Anne Wilson, Hyman B. Muss

**Affiliations:** Author Affiliations: Leigh F. Callahan, Christine Rini, Mary Altpeter, Betsy Hackney, Hyman B. Muss, University of North Carolina at Chapel Hill, Chapel Hill, North Carolina; Arielle Schecher, Anne Wilson, patient advisors, Chapel Hill, North Carolina.

## Abstract

Adding aromatase inhibitors (AIs) to adjuvant treatment of postmenopausal women with hormone-receptor–positive breast cancer significantly reduces cancer recurrence. A common side effect of AIs is noninflammatory joint pain and stiffness (arthralgia) similar to arthritis symptoms. An evidence-based walking program developed by the Arthritis Foundation — Walk With Ease (WWE) — reduces arthritis-related joint symptoms. We hypothesized that WWE may also reduce AI-associated arthralgia. However, the potential for different barriers and facilitators to physical activity for these 2 patient populations suggested a need to adapt WWE before testing it with breast cancer survivors. We conducted qualitative research with 46 breast cancer survivors to explore program modification and inform the development of materials for an adapted program (Walk With Ease-Breast Cancer). Our process parallels the National Cancer Institute’s Research-Tested Intervention Programs (RTIPs) guidelines for adapting evidence-based programs for cancer populations. Findings resulted in a customized 8-page brochure to supplement existing WWE materials.

## Introduction

In 2014, an estimated 232,670 women in the United States received a breast cancer diagnosis (1). Most cancers will be diagnosed in postmenopausal women at an early, highly treatable stage, and most tumors will be hormone receptor–positive (2,3). For women with this tumor type, standard adjuvant (postsurgery chemotherapy and/or radiation) treatment generally includes an aromatase inhibitor (AI) to reduce the chances for cancer recurrence (4,5). Third generation AIs — exemestane (Aromasin), letrozole (Femara), and anastrozole (Arimidex) — are pills taken daily for 5 years, with ongoing scientific consideration of additional years (4,5). Musculoskeletal symptoms — noninflammatory joint pain, stiffness, or achiness (arthralgia) — are common side effects of AIs, with an estimated 33% to 61% of women reporting these symptoms (6–9). When joint symptoms are moderate or severe, they interfere with engagement in physical activity, reduce overall quality of life, and can lead to AI discontinuation or not taking the AI dose as prescribed (7,9,10).

The number of women who are likely to experience these symptoms is substantial. In 2012, there were an estimated 2.97 million female breast cancer survivors, 75% of whom had tumors diagnosed as being hormone receptor–positive; most of these patients were likely to have been prescribed an AI (10). In an aging US population — and breast cancer being largely a disease of aging with 61 years the median age at diagnosis (1) — the number of survivors coping with AI-associated arthralgia will continue to grow. Their quality of life and ability to be physically active during adjuvant treatment may depend on the development of effective behavioral interventions to reduce these musculoskeletal symptoms.

Because AI-associated arthralgia symptoms are similar to those caused by arthritis, we hypothesized that a physical activity program developed by the Arthritis Foundation — Walk With Ease (WWE) (11–14) — could have similar benefits for women on AI therapy. However, the potential for unique psychosocial or medical concerns warranted an investigation of the need to adapt WWE for breast cancer survivors. We describe our process for developing and pilot testing materials to adapt WWE as a precursor to program testing in a randomized controlled trial (currently under way). Our adaptation process parallels guidelines developed by the National Cancer Institute’s (NCI’s) Research-Tested Intervention Programs (RTIPs) (15). We offer this description of our adaptation process as an example of how evidence-based physical activity interventions developed for 1 patient population can be adapted for a new patient population.

## Background

**Walk With Ease (WWE).** WWE is a 6-week physical activity program that is evidence-based in both group and self-directed formats (13). In the interest of testing a program with potential for scalability in clinic settings, we selected the self-directed format for adaptation because it does not entail special facilities, equipment, or personnel. Self-directed WWE is grounded in the social cognitive theory constructs of self-efficacy and outcome expectations (17,18), which are important to encouraging exercise among women with a breast cancer diagnosis (19). Accordingly, the 178-page WWE workbook (*Walk With Ease: Your Guide to Walking for Better Health, Improved Fitness and Less Pain*) (11) includes chapters explaining how walking can reduce joint pain; offering practical advice on how to start and stay motivated to exercise on a daily basis; and providing tools such as self-tests, contracts, and diaries. The goal in WWE is 150 cumulative minutes a week of moderate-intensity walking or the equivalent of 30 minutes a day, 5 days a week.

**NCI’s Research-Tested Interventions Programs (RTIPs).** RTIPs is a portal for program planners and public health practitioners that facilitates access to research-tested interventions and program materials (15). The site includes a searchable database of 150 evidence-based interventions focused on various health-related topics, populations, and settings. To facilitate a systematic approach to program adaptation, the site includes a section called *Using What Works: Adapting Evidence-Based Programs to Fit Your Needs* that provides learning modules and adaptation guidelines. The Table lists the RTIPs 9-step process for guideline adaptation. Our own adaptation process parallels the guidelines developed by RTIPs using a series of iterative steps, with step 9 under way in an ongoing randomized controlled efficacy trial.

**Table:** Overview of Walk With Ease Adaptation Process Guided by NCI’s RTIPs Guidelines

NCI’s RTIPs Guideline	Adaptation Processes
Step 1: Determine the needs of your audience and whether the program addresses those needs.	Research team^a^ reviewed WWE workbook for tone and content relevance to breast cancer survivors on aromatase inhibitor therapy who are experiencing joint symptoms.
Step 2: Review the program and its materials with your intended audience for feedback on its appropriateness.	Semistructured interviews focusing on content needs and material adaptation conducted with breast cancer survivors before and/or after reviewing WWE and WWE-BC materials and doing the walking program (study 1/sample 1 and study 1/sample 2).
Step 3: Define the extent of adaptation needed and potential ways to implement the new program.	Research team^a^, patient advisors, and consultants to the research team reviewed findings from the interviews.
Step 4: Develop “mock-up” versions of the adapted products.	Adapted products produced: 2-page flyer; 12-page brochure; 8-page brochure; 8-page brochure with walking diary attached.
Step 5: Work with expert advisors to ensure that the adapted products maintain the accuracy of the originals.	Expert review by authors L.F.C. and M.A., who were leaders in the study funded by the Centers for Disease Control and Prevention to adapt the group format of WWE to a self-directed format and to conduct a 2-group effectiveness test.
Step 6: Pilot test the adaptation with representatives from your audience.	Pilot tested in survivors aged 65 or older (study 1; results presented elsewhere [20]). Pilot tested further in survivors aged 21 or older (study 2/sample 3). Satisfaction with adapted materials investigated through interviews.
Step 7: Modify and revise the adapted program and products based on pilot feedback.	WWE-BC flyer and brochures were modified after each round of interviews.
Step 8: Implement the program.	NA
Step 9: Evaluate the effectiveness of your adapted program and products.	Ongoing NCI-funded randomized controlled trial to test program and product effectiveness.

Abbreviations: NA, not applicable; NCI RTIPs, National Cancer Institute’s Research-Tested Intervention Programs (http://rtips.cancer.gov/rtips/index.do); WWE, Walk With Ease (evidence-based physical activity program offered by the Arthritis Foundation); WWE-BC, Walk With Ease for Women With a Breast Cancer Diagnosis.

a The research team includes members with expertise in oncology, rheumatology, qualitative research methodology, and intervention research. The team also includes 2 breast cancer survivors who are currently taking an AI and consultants providing additional expertise in intervention research focused on cancer patients and survivors.

## Methods

Semistructured interviews lasting no more than 30 minutes were conducted in a sample of breast cancer survivors, primarily by telephone. Study participants also completed questionnaire items regarding their demographics and medical history. All participants provided written informed consent. The study protocol was approved by the UNC Lineberger Comprehensive Cancer Center Protocol Review Committee and the University of North Carolina (UNC) at Chapel Hill Institutional Review Board.

Our adaptation process was conducted with breast cancer survivors recruited for 2 separate studies (Box) through clinics at the North Carolina Cancer Hospital. The median age in our final sample (N = 46) was 67 years (range, 46–87), most were white (90.5%), and 29% had a high school diploma or less education. Fifty-nine percent had self-reported arthritis. For study 1, our sample was limited to women aged 65 or older; for study 2, the age limit was lowered to 21 or older. Inclusion criteria for both studies were 1) stage I, II, or III breast cancer diagnosis, 2) currently on AI therapy, 3) self-reported joint pain, stiffness, or achiness that is more than mild, 4) engaged in less than 150 minutes of physical activity a week, 5) able to engage in moderate-intensity physical activity, and 6) English speaking. We inquired whether their joint symptoms were recent or of increased intensity in order to identify survivors whose pain, stiffness, or achiness was most likely to be associated with taking an AI.

**Box. Flowchart for Study 1 and Study 2**

**Study 1: Breast cancer survivors on aromatase inhibitor aged 65 or older**

**Sample 1: Formative (N = 10)**

- Interviews only, no walking
- Development of 2-page flyer *Walk With Ease for Women with Breast Cancer on Aromatase Inhibitor (AI) Therapy*

**Sample 2: Pilot test (N = 20)**

- 6-Week walking program
- Postwalking interviews

**Study 2: Breast cancer survivors on aromatase inhibitor aged 21 or older**

**Sample 3: Formative (N = 16)**

- Development of 12-page brochure
- Prewalking interviews
- Refinement of 12-page brochure
- 6-Week walking program
- Postwalking interviews
- Finalization of 12-page brochure

The interview team consisted of the interviewer, note taker, and, depending on their availability, patient advisors and other members of the research team. Immediately following each interview, interview notes were typed up by the note taker, which were reviewed and edited by others who participated in the interview. Typed interview notes were analyzed by 3 research team members; they independently identified themes in the data using thematic analysis, which shares with grounded theory an emphasis on identifying themes that are grounded in the data (20–22). This approach allowed for identification of meaningful themes directly representing participants’ experiences and feedback, without a priori assumptions about those themes. After these initial analyses, team members met to review their findings and inductively derive overarching themes and subthemes across all interview topics. Disagreements were resolved through group discussion to gain consensus.

## Adaptation Process

In our adaptation process (Table), several RTIPs steps are combined, reflecting how the process actually flowed when implemented under the real-world conditions of formative and pre–post evaluation in 2 sequential studies.

### Step 1

The first step is a determination of the needs of the intended audience and whether the program addresses those needs. Our research team includes expertise in oncology, rheumatology, physical activity, qualitative research methodology, health promotion, and intervention research. The team also includes 2 breast cancer survivors who are currently taking an AI (our study’s patient advisors) as well as consultants providing additional expertise in intervention research focused on cancer patients and survivors. The team reviewed the WWE workbook (11) for tone and content relevance to breast cancer survivors experiencing AI-associated joint symptoms and determined that there was a need to explore whether and how WWE should be adapted for our new patient population.

### Steps 2, 3, 4, and 7

In our iterative adaption process, several RTIPs steps were repeated with sequential groups of patients: 1) review the program and its materials with the intended audience for feedback on appropriateness, 2) define the extent of adaptation needed and potential ways to implement the new program, 3) develop mock-up versions of the adapted products, and 4) modify and revise the adapted program and products based on pilot feedback. Completing these steps through 3 sequential samples of survivors (Box) enabled us to conduct a recursive insights-gathering process about participant needs for information and motivational strategies at each juncture and whether larger changes were needed in the WWE program itself.

For study 1/sample 1, 10 survivors aged 65 or older were recruited for a general discussion of 1) likes and dislikes regarding walking for exercise and enjoyment, potential benefits of walking, and challenges or barriers to walking on a regular basis; 2) initial thoughts regarding the WWE program (eg, the goal of 150 accumulated minutes of walking per week, self-directed to let participants determine their own pace at goal achievement); and 3) specific motivations for breast cancer survivors to consider a walking program to reduce their joint symptoms. Content analysis of findings from these formative interviews revealed participants’ desire for supplementary information specific to potential musculoskeletal side effects of AIs and how moderate physical activity might reduce these side effects. Results of this discussion precipitated the team’s development of a 2-page informational flyer titled *Walk With Ease for Women With Breast Cancer on Aromatase Inhibitor Therapy*. Adhering to the self-directed WWE’s basis in social cognitive theory, the flyer’s content was focused on self-efficacy and outcome expectations: 1) general benefits of physical activity for cancer patients and survivors (less fatigue, improved body image, less depression and anxiety, fewer sleep problems, overall improved health and quality of life), 2) potential AI therapy side effects of new or more severe joint pain, and 3) brief overview of the WWE program and how it might relieve their joint symptoms. In addition, participant quotes on the potential benefits of walking, their own motivations to try a walking program, and motivational thoughts for other breast cancer survivors were compiled into a 1-page flyer (*Breast Cancer Survivor Perspectives on Walking for Pleasure or Exercise*). Interview findings also prompted the inclusion of a 6-week walking diary, as both a motivational tool and an additional record of self-reported walking to measure participant adherence to the 6-week walking program.

For study 1/sample 2, a new sample of 20 survivors aged 65 or older were recruited and interviewed after they had completed the walking program to see whether there were additional or different barriers or facilitators to doing the walking program after they had experienced it. Participants received the 2-page informational flyer, 6-week walking diary, 1-page flyer of quotes, and WWE workbook. They were interviewed about their overall experience with the walking program and views on the 2-page flyer (tone and content) and were asked to identify motivational messages that they thought were the most helpful. They were also asked who would be the most effective person for advising or recommending that breast cancer survivors engage in walking (eg, oncologist or oncology clinical personnel, general practitioner), and they were asked about strategies for encouraging breast cancer survivors to become or stay physically active (eg, walking in a group, contact from a professional counselor or breast cancer survivor, methods such as telephone calls, emails, texts, or blogs). Content analyses of findings from these formative interviews precipitated an expansion of the original 2-page flyer into a brochure. Because some participants were confused about “aromatase inhibitor therapy” (they thought that their adjuvant medication was hormone treatment and that AI therapy referred to something new), the title was changed to *Walk With Ease for Women with a Breast Cancer Diagnosis *(*WWE-BC*)*.* The new brochure included additional quotes from study participants, summarized findings from the pilot study (20), and concluded with a 1-page “My Walking Plan” as an additional motivational tool (walking start date, walking goals, reasons for walking, best days and times to walk, walking buddies, and “what I will tell myself to stay motivated on days when walking is hard”). The ensuing 12-page brochure (developed with desktop publishing software) included inspirational photos of diverse women and had an easy-to-read type size. Although the actual walking experience of sample 2 participants varied (20), interview responses were supportive of our overall approach, which asked women to achieve 150 minutes per week over 6 weeks; the respondents also indicated that the overall program was safe, feasible, and enjoyable.

For study 2/sample 3, 16 survivors from a wide age range — 21 or older — were recruited and interviewed both before and after the walking program. Prior topics were revisited to see if any new information and themes would emerge from this cohort. We asked about 1) participants’ knowledge of AI side effects before receiving the study materials and learning about the objectives of the study, 2) the value of having a discussion with the oncologist about AI side effects and engaging in moderate physical activity to reduce joint symptoms, and 3) their advice on methods for encouraging survivors to attempt and sustain a walking routine. There were no new findings regarding the WWE-BC materials or program; however, there were many findings to inform a future dissemination and implementation study. In addition, findings led us to edit the brochure into a tighter 8-page format that is being tested in our ongoing randomized controlled trial (RCT).

### Step 5

RTIPs guidelines recommend working with expert advisors to ensure the adapted products maintain the accuracy of the originals. Two members of the research team (L.F.C. and M.A.) were principal investigator and co-investigator for a study funded by the Centers for Disease Control and Prevention to adapt the group format of WWE to a self-directed format and conduct a 2-group effectiveness test with a large sample of community-based adults (12–14). They ensured that while adaptation modifications made clarifications for breast cancer survivors, the intervention content remained true to the original WWE health education, exercise, and motivational tool core components.

### Step 6

RTIPs guidelines recommend pilot testing with representatives from the target audience. The initial adaptation of the program was pilot tested with study 1/sample 2 breast cancer survivors aged 65 or older. The program was found to be feasible (walking minutes increased between baseline and 6 weeks), safe (no adverse events), and showed promising effects on joint stiffness (20). In postwalking semistructured interviews, participants reported that the program’s emphasis on walking safely and comfortably and achieving the goal of 150 minutes at each participant’s own pace made WWE-BC enjoyable and realistic, even for women who were largely sedentary.

### Steps 8 and 9

The final 2 steps pertain to implementing the adapted program and evaluating its effectiveness. WWE-BC is still in the efficacy testing phase through an ongoing RCT. In the event findings from the RCT are positive, plans include effectiveness testing in an implementation study.

## Discussion

We describe the adaptation of an evidence-based arthritis program for a new patient population — breast cancer survivors on AI therapy who are experiencing joint symptoms. Our adaptation process paralleled guidelines recommended by NCI’s RTIPs. We found that the RTIPs guidelines are realistic and practical to implement in a real-world context and that they serve as a helpful checklist to ensure that program adaptation is conducted systematically and thoroughly.

The materials we developed to adapt the WWE arthritis program were reviewed and revised through an iterative process involving 46 breast cancer survivors as well as our patient advisors and outside consultants. A limitation of our study is our largely white and well-educated sample, which is not representative of the larger population of breast cancer survivors. However, our interviews did not reveal differences in perspectives or informational needs between white survivors and survivors of other races. It is an empirical question whether responses from a more diverse sample would have substantially changed our findings.

Our adaptations were confined to essential new information, readability, wording, quotes, photos, and ways to reach our target audience. The underlying theory, targeted health behavior, timeline, dosage, and core components remained unchanged, and no new strategies were added that would detract from the core components. This description of our adaptation strategies and process may be informative for others seeking systematic guidance for adapting, implementing, and testing evidence-based physical activity interventions for cancer patients and survivors.
